# PUTTING THE 4MS INTO PRACTICE: IMPLICATIONS FOR TRAINING AND TECHNOLOGY

**DOI:** 10.1093/geroni/igac059.930

**Published:** 2022-12-20

**Authors:** Jennifer Crittenden, Rachel Coleman, Jennifer Jain, David Wihry, Lenard Kaye, Susan Wehry, Judith Metcalf

**Affiliations:** UMaine Center on Aging, Bangor, Maine, United States; University of Maine, Bangor, Maine, United States; University of Maine, Bangor, Maine, United States; University of Maine, Orono, Maine, United States; University of Maine, Bangor, Maine, United States; University of New England, Biddeford, Maine, United States; University of New England, Biddeford, Maine, United States

## Abstract

The AgingME Geriatrics Workforce Enhancement Program (AgingME GWEP), a statewide collaboration led by the University of New England and the University of Maine, is guided by an annual statewide survey of community and professional stakeholders. The aim of this research is to identify training and resource gaps related to age-friendly healthcare and topics of interest. Of the 245 survey respondents, 15% indicated existing knowledge of the 4Ms framework. The top sources of 4M’s framework exposure included trainings or webinars (30%) and web-based resources (19%). Of those with knowledge of the 4M’s, 33% of providers and 29% of older adults/community members reported employing the 4Ms in their professional practices and personal lives, respectively. Respondents also noted the need for more training on how to use technology to locate healthcare information (33%), using technology to reduce isolation and loneliness among older adults (29%), and keeping providers connected with older patients (26%). Additional write-in responses (11%) suggest a need for general technology training and improving access to technology overall. Respondents' (N = 157) top five categories for needed aging-related training topics were community resources for older adults (15%), aging-in-place (14%), exercise and nutrition (11%), improving provider/patient communication (9%), and 4M’s of age-friendly healthcare overview (8%). Responses also identified themes related to improving patient/provider communications, availability of resources, and ageism that could be addressed through upcoming GWEP activities. Results indicate a need to facilitate the translation of 4Ms knowledge into practice and increasing technology training and access.

